# Following the fate of polystyrene micro and nanobeads during *in vitro* digestion

**DOI:** 10.1016/j.crfs.2025.101086

**Published:** 2025-05-19

**Authors:** Elena Arranz, Emmanouil D. Tsochatzis, Negin Hashemi, Hanne Søndergaard Møller, Milena Corredig

**Affiliations:** a Sección Departamental de Ciencias de la Alimentación, Facultad de Ciencias, Universidad Autónoma de Madrid (UAM) & Instituto de Investigación en Ciencias de la Alimentación, CIAL (CSIC-UAM, CEI UAM + CSIC), Nicolás Cabrera, 9, 28049, Madrid, Spain; b Department of Food Science, Aarhus University, Agro Food Park 48, 8200, Aarhus N, Denmark

**Keywords:** Polystyrene nanoparticles, *In vitro* digestion, *In vitro* cell permeability, GS-MS analysis

## Abstract

The objective of this project was to follow the fate of nano and microplastic particles during gastrointestinal transfer. Polystyrene latex nano (60 nm) and micro (1 μm) beads were employed as model system for microplastic particles and mixed (68 mg/mL) with 5 % (w/v) whey protein solution to study the food effect. The digestion of this mixture was then subjected to the INFOGEST *in vitro* digestion protocol. Residual particles in the digesta were then loaded on 21 days differentiated co-cultures of Caco-2/HT29-MTX, to further determine their potential transfer through intestinal monolayers. Digested samples diluted 1:16, showed no cytotoxic effect on Caco-2 cells, possibly due to the presence of a protective mucus layer. Digestion and permeability experiments were further analysed using gas chromatography with mass spectrometry (GC-MS), and different monomers (styrene), styrene oligomers (1-phenyl-1,2-ethanediol; 1,4-diphenyl-1,3-butadiene; 1,2-diphenylcyclopropane; α-methyl benzenemethanol), other intentionally added substances (caprolactam, benzaldehyde, di(2-ethyl hexyl) phthalate) and non-intentionally added substances (e.g. 2,4-di-tert butylphenol, isophthalaldehyde) were identified. Analysis of the basolateral fraction indicated a notable transfer of these compounds through cell membranes. This holistic approach using a food matrix to follow the fate of microplastic during digestion leads to a better understanding of the risks of microplastics and nanoplastics through food.

## Highlights

• Evaluation of nano- and microplastic fate in gastrointestinal transfer.
• Cytotoxicity of digested nano- and microplastic particles.
• Intestinal permeability tested in Caco-2/HT29-MTX cell cultures.
• Identification of styrene monomers, oligomers and polymer additives.

## Graphical abstract

**Figure:**
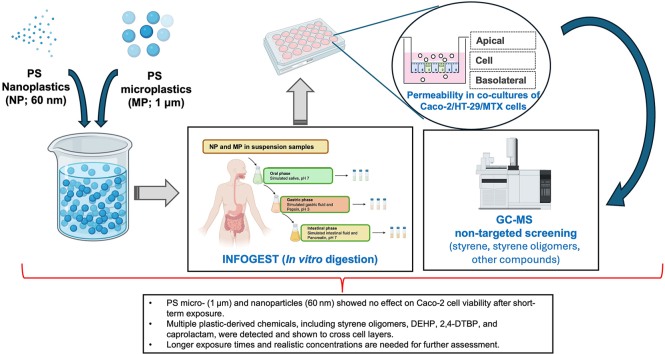

## 1 Introduction

The increasing production and use of plastic have led to growing concerns regarding microplastics (MPs) and nanoplastics (NPs) occurrence in the environment, in food and water. MPs or NPs are of emerging environmental concern. Given their small size, MPs could be ingested by humans through contaminated food, water, and air, raising concerns about their potential health impact. These particles could be derived, for example, from abrasion or mechanical disruption, or through aggregation of insoluble molecules (Wang et al., 2024; Yang et al., 2024). MPs and NPs can originate from intentional applications or from unintended sources, including polymer degradation from packaging and environmental pollution (Ivleva, 2021; Zhang et al., 2024; Fang et al., 2023). MPs are defined as plastic particles between 1 μm and 5 mm in size (EFSA Panel on Contami nants in the Food and Chain (CONTAM), 2016), whereas NPs of less than 1 μm. The latter may be even more challenging due to their higher bioavailability and potential to translocate into biological tissues.

Recent advances in analytical chemistry and toxicological assessments have enabled more precise detection and quantification of MPs and NPs in various biological and environmental matrices, highlighting the need for harmonized methods to assess their presence, fate, and potential toxicity. Additionally oligomers may be generated through incomplete polymerization or degradation of the polymers; these compounds are a few nanometers in size as indicated by computational chemistry, for polystyrene (PS), polyethylene terephthalate (PET), and polybutylene terephthalate (PBT), and may pose a potential risk by being able to penetrate biological barriers (Tsochatzis et al., 2023, 2024).

The gastrointestinal tract may serve as a major entry point for MPs, NPs, and their associated chemical oligomers. Research suggests that smaller NPs and oligomers can be transmitted through the intestinal barrier via various mechanisms, including transcellular transport and endocytosis. Once absorbed, these compounds may circulate in the bloodstream and accumulate in different tissues, potentially leading to toxic effects. Given their ability to cross biological membranes, their impact on cellular function and metabolism remains a critical area of research (Tsochatzis et al., 2023, 2024). However, their fate through gastrointestinal digestion and transit has yet to be described in detail.

In this work, PS was used as a model as it is recognized as a potentially carcinogenic compound (Styrene and Styrene, 2019; EFSA Panel on Food Contact Materials et al., 2020). During environmental weathering or ageing, PS could release residual monomers, oligomers, and additives, such as styrene monomer, up to a concentration of 0.17 μg/mL within 24 h (Björnsdotter, 2015). Additionally, additives and non-intentionally added substances (NIAS) may also leach from the PS material, contributing to their overall toxicity (Bridson et al., 2023). Recent studies investigated cytotoxic and inflammatory responses induced by PS-MPs in various cell lines. For example, research using human lung epithelial BEAS-2B cells demonstrated that PS-MPs can cause cytotoxicity and inflammation. Their presence disrupted the pulmonary barrier by depleting tight junction proteins, potentially increasing the risk of respiratory diseases (Dong et al., 2020). Another study highlighted that standard PS particle dispersions, containing residual monomers, exerted low but significant cytotoxicity on mammalian cells (Zhang et al., 2023).

A holistic study which follows the fate of PS micro and nanoparticles in food during digestion is needed. A well-established *in vitro* model system to study the intestinal permeability of food components or pollutants is differentiated Caco-2 cells, grown in Transwell® plates (Hubatsch et al., 2007). Co-cultures with Caco-2 cells and mucus producing cells, HT-29/MTX cells, are more physiological models that allow studying the effect of the mucus layer (Lozoya-Agullo et al., 2017). Caco-2 cells, which differentiate into enterocyte-like cells, model the small intestinal epithelium, while HT-29/MTX cells mimic mucus-producing goblet cells, providing a physiologically relevant co-culture system. These models allow for studying MP/NP uptake, translocation, cytotoxicity, oxidative stress, and inflammation in the gut (Herrala et al., 2023; Soto-Bielicka et al., 2024; Chen et al., 2023; Marcellus et al., 2025), especially when following *in vitro* digestion.

This study aims to utilize PS particles of defined size to evaluate their fate during *in vitro* digestion and absorption. An established *in vitro* model system for digestion (INFOGEST), was employed on a mixture of PS with whey protein suspension, to assess if gastrointestinal transit may influence the PS particles. The permeability of PS through differentiated monolayer co-cultures of Caco-2 and HT-29/MTX was then evaluated, by analyzing mucus layer, cells and basolateral fractions using GC-MS. This work will ultimately provide insights on the potential role of food as a vector for exposure of environmental microplastic.

## 2 Materials and methods

### 2.1 Chemicals

Dichloromethane (DCM; CAS: 75-09-2) were Chromasolv grade purity and obtained from Sigma Aldrich (Steinheim, Germany). Ultra-pure water (18.2 MU) for the preparation of food simulant A (10 %v/v ethanol in water) and D1 (50 % v/v ethanol in water) respectively), was obtained with a Milli-Q system (Millipore, Bed-ford, USA). Polytetrafluoroethylene (PTFE) 17 mm, 0.2 mm membrane filters were supplied by CPS Analitica (Milan, Italy). Target analytes were supplied by Sigma Aldrich, FUJIFILM Wako Chemicals Europe GmbH (Milano, Italy) and TRC Chemicals (Toronto, Canada). In Table 1 are presented the most relevant analytes characteristics, while molecular structures are given in Fig. S3 (see Supplementary material). Polystyrene latex particles (PS) of 60 nm and 1 μm (10 % w/v) were supplied by MAGSPHERE INC (Pasadena, California, USA). Whey protein isolate (WPI, protein content 90 % w/w) was purchased from Myprotein Impact (Denmark). All other reagents were purchased from Sigma-Aldrich unless stated otherwise.

**Table 1:** Identification of structurally similar compounds to styrene by GC/MS analysis.

No.	Retention time (min)	Identified compound^a^	Chemical formula	Molar mass (Da)	Monoisotopic mass (Da)	Molar ion m/z	Abundant ion (m/z)
1	2.362	Styrene	C8H8	104.1	104.0	104.0	104.0
2	2.807	Benzaldehyde	C7H6O	106.1	106.0	106.0	105.1
3	2.946	4-methyl-benzaldehyde	C8H8O	120.1	120.1	120.1	119.0/91.1
4	3.352	α-methyl benzenemethanol (or 1-phenylethanol)	C8H10O	122.2	122.2	122.2	107.0
5	3.744	4-methyl benzaldehyde	C8H8O	120.1	120.1	120.2	105,1
6	4.010	Caprolactam	C6H11NO	113.0	113.0	113.1	113.0/55.0
7	4.177	1-phenyl-1,2-ethanediol	C8H10O2	138.1	138.1	138.1	107.1
8	4.195	α-2-propenyl-benzenemethanol (or 1-Phenyl-3-buten-1-ol)	C10H12O	148.2	148.1	148.1	107.0
9	4.336	Nonanal	C9H18O	142.2	142.1	142.1	57.0
10	4.963	2,4,-ditert-butyl phenol (2,4-DTBP)	C14H22O	206.3	206.2	206.2	191.2
11	6.799	1,4-diphenyl-1,3-butadiene	C16H14	206.3	206.1	206.1	206.1
12	7.697	1,2-diphenylcyclopropane	C15H14	194.3	194.1	194.1	193.1/115.0
13	7.916	α-hydroxy-2-benzeneacetic acid) (or Mandelic acid)	C8H8O3	152.1	152.0	152.1	107.0
14	9.013	Isophthalaldehyde	C8H6O2	134.0	134.0	134.0	133.0
15	12.818	DEHP	C24H38O4	390.6	390.3	390.2	149.0

a **NOTE:** Identification was based on MS spectra matching using NIST 2014 and confirmed with ACD/Labs MS workbook software.

### 2.2 Preparation of polystyrene suspensions and simulated gastrointestinal digestion static model

Four formulations were prepared, two PS formulations containing 6.8 % w/v of 0.06 or 1 μm PS particles and two PS mixture with the same PS concentrations but also containing 5 % w/v of WPI (WPI-PS) in Milli-Q H_2_O. The four treatments were then subjected to simulated *in vitro* gastrointestinal digestion following the standardised INFOGEST method with minor modifications (Brodkorb et al., 2019), in triplicate. The oral phase was simplified to no amylase addition and dilution of 2.5 mL of liquid formulation with 2 mL of simulated salivary fluids, 12.5 μL CaCl_2_ 0.3 M and Milli-Q H_2_O up to 5 mL. The gastric phase followed the dilution in the oral phase with the addition of 4 mL of simulated gastric fluids (SGF), 2,5 μL of CaCl_2_ 0.3 M and 0.333 mL of porcine pepsin (EC 3.4.23.1, dissolved in H_2_O to reach a final concentration of 2000 U/mL, activity 362 U/mg). The pH was adjusted to 3 with 1 M HCl, Milli-Q H_2_O was added to reach 10 mL final volume and samples were incubated at 37 °C in a rotator disk at 15 rpm for 2 h. After the incubation, the intestinal phase followed with the addition of 20 μL CaCl_2_ 0.3 M, 4 mL simulated intestinal fluids (SIF) containing porcine bile (10 mM in the final mixture, concentration of bile salts 1331.6 μmol/g), 4 mL of SIF with pancreatin (EC 232.468.9, to reach a final concentration of 100 U/mL, activity 8.7 U/mL). The pH was increased to 7 with 1 M NaOH and Milli-Q H_2_O was added to adjust the final volume to 20 mL. Samples were incubated at 37 °C in a rotator disk at 15 rpm for 2 h. A control *in vitro* digestion substituting the sample with Milli-Q H_2_O was also run in parallel. After the intestinal phase, the enzyme activity was stopped by snap freezing the samples on liquid nitrogen and subsequently stored at −20 °C until further analysis.

### 2.3 Protein profiling of WPI-PS formulations by SDS-PAGE

Samples containing WPI were analysed through SDS-PAGE before and after digestion. Each sample was mixed with 2.5 μL of NuPAGE® LDS Sample buffer (4X) (ThermoFisher, Denmark) and 1 μL NuPAGE reducing agent (ThermoFisher). The prepared samples were heated for 5 min at 95 °C. Aliquots (10 μL) of each sample were loaded onto 4–12 % NuPAGE Bis-Tris gel and NuPAGE® MES-SDS buffer (20X) (ThermoFisher) was used as running buffer. The separation process was performed at 200 V for 45 min. After separation, the gel was stained with SimplyBlue™ SafeStain (ThermoFisher).

### 2.4 Confocal laser scanning microscopy of digesta

Confocal laser scanning microscopy (CLSM) was carried out using a Nikon microscope (Nikon AX, Nikon Instrument Inc, Tokyo, Japan) equipped with a 10X objective to visualize protein distribution in SGID WPI-PS samples. For fluorescent labelling 0.1 % (w/w) of FITC green for protein was used. The fluorescent dye was dissolved in acetone and a droplet of the mixture was applied to a cover slide. After acetone evaporation, the samples were placed onto the cover slide. FITC green was excited at a wavelength of 488 nm while observation was made at emission wavelengths of 505–551 nm.

### 2.5 Cumulative effect in Caco-2 cells and permeability assay in co-cultures of Caco-2/HT-29/MTX cells

Caco-2 and HT-29/MTX cells were obtained from the Tissue Culture collection of the Department of Food Science, Aarhus University (Denmark). Cells were grown separately in 75 cm^2^ tissue culture flasks with culture medium in an incubator at 37 °C and 5 % CO_2_ humidified atmosphere. Cells growth medium contained 10 % (v/v) FBS, 1 % (v/v) nonessential amino acids, 1 % (v/v) antibiotic solution (penicillin–streptomycin), and 1 % (v/v) sodium pyruvate. The cell culture medium was replaced every two days and cells were sub-cultured with 0.5 % trypsin-EDTA when 80 % cell confluency was reached. The cells were ready for use after three sub-cultures. Caco-2 and HT29-MTX cell in this study were used at passage number 10–15 and 9–12, respectively.

Before cumulative assays, the cytotoxicity of undigested PS suspensions was determined in Caco-2 cells by MTT assay. The cytotoxicity assay was conducted only on Caco-2 cells, and not on HT29-MTX cells, as the latter are known to be more resilient to a wide range of different compounds including polyphenolic compounds, protein and fat substances, primarily due to the protective effect of the mucus layer they produce (Guri et al., 2014; Kondrashina et al., 2023; Hashemi et al., 2025). Caco-2 cells were seeded in 96-well plates at a concentration of 8 × 10^4^ cells/well and after 24 h were washed twice with PBS. Undigested PS suspensions were added to each well at the following concentrations, 25, 50, 100, 250 and 500 μg/mL in complete DMEM for 24 h. After 24 h, the treatment was discarded. Cells were washed twice with PBS, then 10 μL of MTT solution (5 mg/mL in PBS) and 100 μL of complete DMEM were added to each well and incubated for 4 h. After incubation, the medium was removed and 150 μL of DMSO were added to each well. The formazan produced was determined by measuring the absorbance at 570 nm in a microplate reader (Synergy 2, BioTek, Fiedrichshall, Germany). Results were presented as the percentage of cell viability, calculated as the absorbance of the sample relative to the absorbance of the control (cell with medium only), and six replicates were performed.

Similarly, before permeability assays, the cytotoxicity of SGID PS and WPI-PS formulations was determined in Caco-2 cells by MTT assay. SGID samples were diluted, 1:16 sample:DMEM complete medium, containing 500 μg/mL of 1 μm or 60 nm PS particles to treat 24 h confluent Caco-2 cells in 96-well plates for 4 h. After 4 h, cells were treated as described previously to determine the formazan produced. Six replicates were performed.

The cumulative effect of undigested 1 μm or 60 nm PS particles without WPI was assessed in confluent Caco-2 cells. Cells were seeded at 1 × 10^5^ cells/well in 24-well plates and incubated for 24 h. After 24 h, cells were washed twice with PBS and 500 μg/mL of PS in complete DMEM was added to the wells. Cells were exposed for 24, 48 or 72 h, replacing the media with fresh PS suspension every 24 h. After each 24, 48 or 72 h time, cells were observed using an optical microscope and images were taken. Then, cells were harvested with trypsin and viable cells were counted after Trypan Blue dilution with a Neubauer chamber. The cumulative effect was determined in triplicate.

Permeability assays were performed in 24-well Transwell® plates (0.4 μm pore size polycarbonate, surface area of 6 × 10^−5^ m^2^ (Merck-Millipore, Darmstadt, Germany)). Caco-2 and HT-29/MTX were seeded together (co-cultured) at a ratio of 75:25, respectively, at a final density of 1 × 10^5^ cells per insert in complete DMEM. Every two days the cell culture media was replaced, and cells were incubated and allowed to differentiate for 21 days. On day 20 and 16 h before the permeability experiment, the culture media was replenished to prevent cell starvation. On treatment day (day 21), apical and basolateral compartments were washed three times with HBSS. Monolayer integrity was determined by means of transepithelial electrical resistance (TEER) (Ω•cm^2^) using a Millicell-ERS Voltohmmeter, TEER values below 600 Ω cm^2^ were discarded. Then, 400 μL of SGID PS and SGID WPI-PS samples diluted 1:16 in complete DMEM were added to the apical compartment and 600 μL of complete DMEM to the basolateral compartment. Undigested PS suspensions of 1 μm or 60 nm PS particles were also tested and added to the apical compartment at the same dilution rate. Plates were incubated for 4 h at 37 °C with a 5 % CO_2_ humidified atmosphere and TEER measurements were noted at times 0 and 4 h. A control sample with HBSS was also run in parallel. After incubation, cells were harvested with trypsin and apical and basolateral contents were collected and stored at −20 °C until further analysis. Each SGID sample was assayed in quadruplicate per SGID replicate.

### 2.6 Extraction

A liquid-liquid extraction (LLE) method was applied following a previous published method (Tsochatzis et al., 2020a). In brief, a sample volume of 1.5 mL of the sample (digesta) was placed together with of 1.5 mL of dichloromethane (DCM) was added to a tube. The tube was vortexed for 1 min and centrifuged using an Eppendorf 5810 R refrigerated centrifuge, set at 20 °C and 2500 rpm (1750 *g*) for 5 min. The procedure was repeated for a second time by adding 1.5 mL of DCM. The DCM extracts (bottom solvent layers) were combined, filtered with PTFE 0.22 mm filters and evaporated to a final volume of 1.5 μL. A volume of 1 μL, from the final DCM solution, was injected to the GC-MS system. Two replicates per SGID sample were analysed.

### 2.7 GC-MS

Chromatographic analyses were performed based on a previous analytical method (Tsochatzis et al., 2020a), with a GC (Agilent Technologies 5975 GC) equipped with a single quadrupole mass detector (Agilent Technologies 5975 MSD) at a constant flow rate of 1.2 mL min^−1^ of helium. The chromatographic column (HP- 5MS UI 5 % phenyl methyl siloxane, 15 m × 250 mm, 0.25 mm) was supplied by Agilent Technologies Inc. (Agilent Technologies, USA). The analysis was per-formed using a split/splitless injector at 250 °C, operating in the splitless mode. The used injection volume was 1 μL. The oven program was the follow: initial temperature of 50 °C for 1 min, ramp (40 °C min^−1^) up to 150 °C (isothermal of 1 min), ramp (15 °C min^−1^) up to 300 °C (isothermal of 1 min), ramp to 325 °C (25 °C min^−1^) and a final hold time of 1 min. The total run time was 15.5 min, including a solvent delay time of 3 min. The detection was performed with positive Electron Impact (EI) at 70 eV, and the resulting chromatograms were processed using Extracted Ion Chromatogram (EIC) mode of the software.

### 2.8 GC-MS data and spectral analysis

All GC-MS analytical data was processed with Masshunter 10.0 software (Agilent Technologies, USA). The obtained analytical data were further Mass Profiler Professional (MPP) v.15.0 software (Agilent Technologies, USA) for alignment and exploitation of the received MS data. For the identification of compounds, NIST 2014 library was used. MPP is the only platform that provides integrated identification/annotation of compounds and automated sample classification in combination with Masshunter. MS data were further processed with the ACD/Labs software (ACD/Labs, Toronto, Canada) in combination with Agilent's MassHunter (Agilent Technologies) data, in order to assess and evaluate all untargeted screening data (e.g. chromatographic and MS data). This software allows the confirmation and checking of the consistency of any suggested chemical structures MS spectra, as well as assigning experimental spectra and molecular ion or generated fragments to structures by comparing experimental MS data with theoretical MS and MS fragmentation data.

### 2.9 Statistical analysis

Statistical analysis of replicate samples was performed using Microsoft Excel to establish and confirm the significance of important identified variables. Furthermore, a multivariate statistical analysis was performed using Simca-P v17.0.2 software (UMETRICS AB Sweden). Orthogonal Partial Least Squared Discriminant Analysis (OPLS-DA) was used for modelling the differences between the different tested samples, as well as their statistical significance and model fit and reliability, where the R^2^Y and Q^2^ approach was applied. Variable importance for prediction (VIP) scores were also calculated for the OPLS-DA models aiming to identify potential markers or compounds discriminating the tested set of data.

## 3 Results and discussion

### 3.1 Cytotoxicity and cumulative effect of polystyrene suspensions in Caco-2 cells

**Fig. 1:**
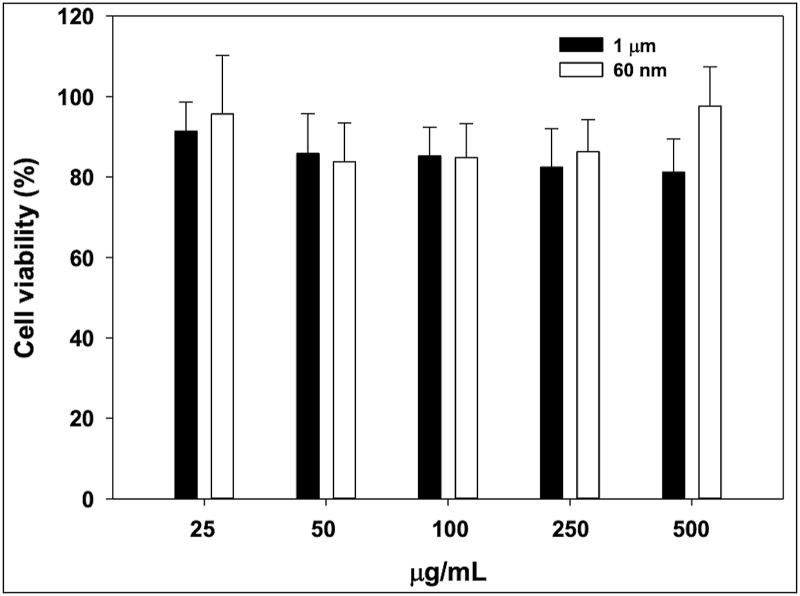
Cell viability (%) of confluent Caco-2 cells after 24 h incubation with 1 μm or 60 nm polystyrene latex particles, 25–500 μg/mL diluted in DMEM complete medium. Control cells were grown in DMEM complete medium with no treatment (100 % viability). Values are the average of six replicates; bars represent standard deviations.

The cytotoxicity of undigested PS suspensions was tested on confluent Caco-2 cells. Fig. 1 shows the results after 24 h exposure of 25–500 μg/mL of PS, measured by MTT assay. The 25 μg/mL concentration was chosen based on being one hundred times more than the average reported intake of microplastics in humans at 0.166 g/day (Tamargo et al., 2022) and an approximate surface of the human gut mucosa of 200 m^2^. None of the tested concentrations significantly decreased cell viability after 24 h exposure compared to 100 % viability of cells with complete medium. The size difference, for the 1 and 0.06 μm PS suspensions, showed no significant differences. Due to their lack of cytotoxicity, the high concentration of 500 μg/mL was chosen to test the effect of cumulative exposure in Caco-2 cells to PS plastic beads, as a worst-case scenario.

**Fig. 2:**
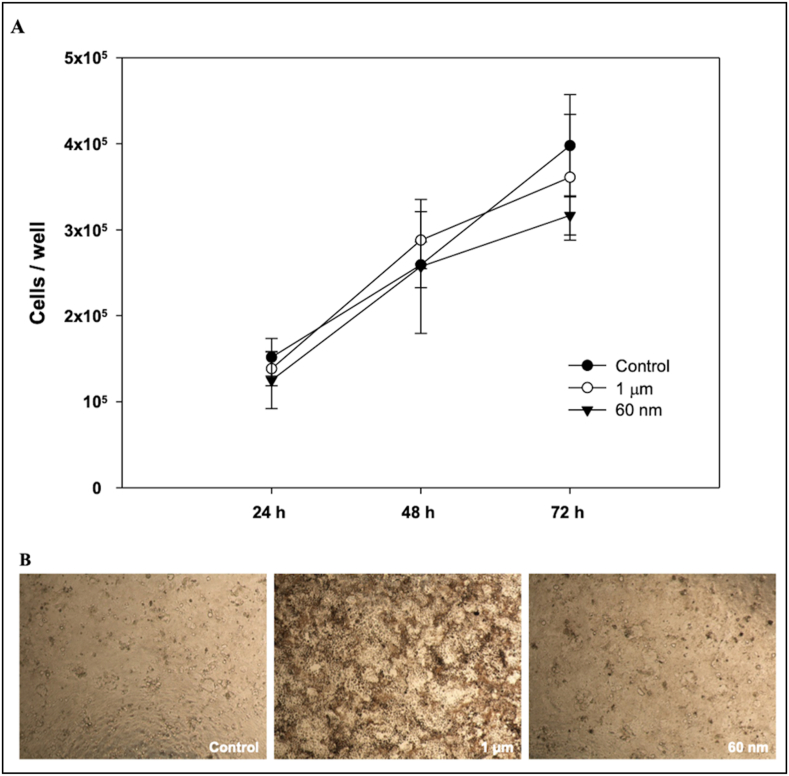
Caco-2 cell count per well (A) in 24 well/plates after cumulative exposure to 500 μg/mL of 1 μm or 60 nm polystyrene latex particles during 24, 48 or 72 h. Control cells were grown in DMEM complete medium with no treatment. Culture media and treatments were refreshed every 24 h. Values are the average of three replicates; bars represent standard deviations (B) Optical microscope images of Caco-2 cells after 72 h treatment (4x objective).

Fig. 2 summarizes the effect of the PS beads suspensions on Caco-2 cells viability, after 24, 48 and 72 h of exposure. The presence of PS suspensions, regardless of the size, did not affect cell growth, during exposure (Fig. 2A). There were no statistically significant differences at 24, 48 or 72 h compared to cells grown in control medium. Images collected with optical microscope (Fig. 2B) show the presence of 1 μm PS particles in the cells' cytoplasm after 72 h, demonstrating their absorption. The 0.06 μm PS particles were not visible at this magnification. These results supported what already extensively reported in the literature; for example, Saenen et al. demonstrated the uptake of 100 μg/mL of 200 nm and 2 μm PS particles in confluent Caco-2 cells after 24 h exposure (Saenen et al., 2023). Moreover, the authors demonstrated cell membrane damage with the 2 μm PS particles but not with the 200 nm PS particles. Wang et al. reported that PS particles of 300 nm to 6 μm at a concentration of 20 μg/mL were presented inside the cytoplasm of Caco-2 cells after 24 h exposure (Wang et al., 2020). A long-term effect of 0.26 μg/cm^2^ PS exposure in Caco-2 cells, demonstrated that the levels within the cell remain stable during 8 weeks (Domenech et al., 2021).

### 3.2 Effect of the simulated gastrointestinal digestion (SGID) on WPI polystyrene suspensions

Four treatments were digested, two containing only PS particles (0.06 or 1 μm), and two with PS particles mixed with a whey protein solution (5 % w/v of WPI). The suspensions were then subjected to simulated gastrointestinal digestion using the standardised INFOGEST protocol. The addition of WPI in PS suspensions aimed to explore the effect of a food product as being one of the main pathways to how polystyrene may enter the human body. Controls with 5 % w/v of WPI solution in water and H_2_O as a blank were also included in the experimental design.

**Fig. 3:**
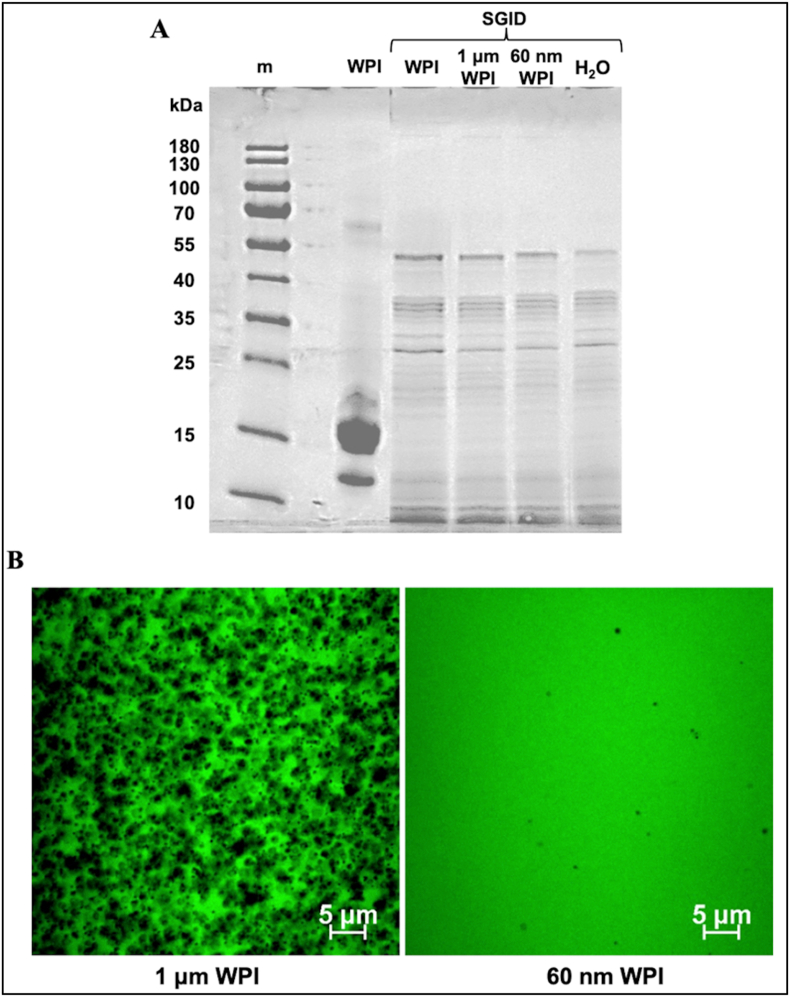
Reducing SDS-PAGE protein profile (A) before and after simulated gastrointestinal digestion (SGID) of whey protein isolate (WPI) and SGID WPI formulations containing 1 μm or 60 nm polystyrene latex particles. A control sample with H2O was also subjected to the same SGID process. CLMS micrographs (B) of digested WPI formulations containing 1 μm or 60 nm polystyrene latex particles. The green colour represents protein and the scale bars are 5 μm.

To monitor WPI digestion, protein profiling was performed in WPI-PS formulations and WPI suspension by SDS-PAGE, Fig. 3A. The results showed that WPI formulations and WPI in water had a similar protein digestion profile, with the only visible bands corresponding to digestive enzymes, mainly pancreatin. The same protein profile was observed in all digested samples, including the WPI and H_2_O controls. The addition of PS particles did not interfere with WPI digestion. WPI was fully digested into small peptides with a similar pattern of bands. The digested samples were also assessed using confocal microscopy. WPI-PS formulations (Fig. 3B) showed that 1 μm particles formed aggregates after digestion, of almost 5 μm (dark aggregates). On the contrary, WPI-PS digesta containing 0.06 μm showed a more homogenous distribution, with a small distribution of aggregates, of sizes less than 2 μm. It is expected that PS particles would not undergo digestion. This has been already demonstrated in the literature. For example, Lui et al. reported no chemical alterations for PS particles of 100 nm and 5 μm, although a protein corona forms on the surface of the particles (Liu et al., 2020; Brouwer et al., 2024). The aggregation noted in our work is in full agreement with these results.

### 3.3 Permeability of polystyrene particles in differentiated co-cultures of Caco-2 and HT-29/MTX cells

Co-cultures of Caco-2 and HT-29/MTX cells were differentiated for 21 days and exposed to the gastrointestinal digesta, namely those containing 0.06 or 1 μm PS particles, with or without WPI, as well as a H_2_O control, undigested 0.06 or 1 μm PS particles and a buffer blank (HBSS). Permeability assays were performed at a concentration of 500 μg/mL of PS particles for 4 h. SGID samples were diluted in DMEM complete medium (1:16) to reach a concentration of 500 μg/mL of PS particles, dilution that did not cause cytotoxicity (see Fig. S1, Supporting Information). On day 21, an average TEER value of 1606 ± 129 Ω cm^2^ was obtained at time 0 h. After 4 h of incubation, the TEER results (see Fig. S2, Supporting Information) show no significant differences between all samples. Therefore, it could be concluded that the monolayer integrity, by means of transepithelial resistance, was not altered. These results are in contrast to what was reported in the past. Liu et al. showed that 20 μg/mL of undigested 100 nm PS particles significantly increased lucifer yellow permeability in Caco-2 differentiated monolayers, causing intestinal transport damage (Liu et al., 2020). This discrepancy could be attributed to the formation of a corona from proteins or proteases, and more importantly to the presence of the mucus layer synthetized by the HT-29-MTX cells, which mimics closer the human gut and might protect from permeability disruptions caused by PS particles.

### 3.4 Identified compounds

**Fig. 4:**
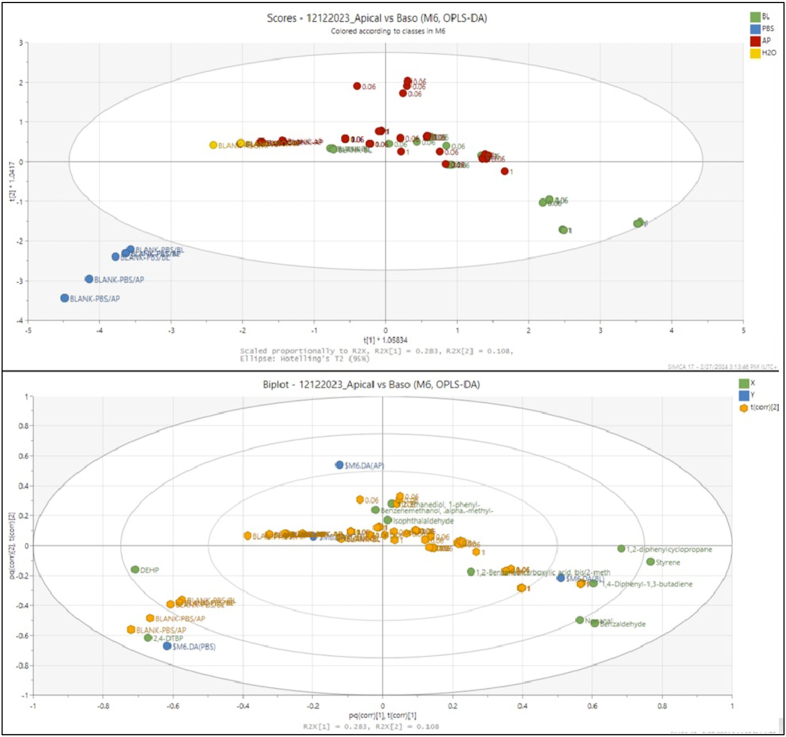
OPLS-DA biplot for the different treatments in the analysed apical/basolateral fractions for the identified styrene and styrene-based compounds.

After absorption experiments, samples were collected from the apical, cells and basolateral fractions, to evaluate their transport through the simulated intestinal monolayer. The fractions were analysed by GC-MS and a range of chemical compounds structurally related to styrene was identified based on MS library matching with the NIST2014 library (NIST, USA), and after *in silico* confirmation with ACD/Labs MS Workbook Chemoinformatic tool. The compounds identified are given in Table 1. It is important to note that due to the limited exposure time into the cell membrane and due to the low volume, certain metabolites might have been below the analytical method's limit of detection (LOD). Multivariate analysis (MVA) was performed for the identified compounds as reported in Table 1 and Fig. 4.

Styrene as a monomer can be oxidized to form styrene oxide acetophenone, which can be considered one of the basic side reaction products (EFSA Panel on Food Contact Materials et al., 2020; Tsochatzis et al., 2021a). However, styrene oxide is a very volatile compound and can be easily eliminated in simple laboratory environmental conditions (temperature). 1-phenyl-3-butan-1-ol and 1-phenyl-1,2-ethanediol can be considered as styrene structurally related compounds. They can be potentially generated from biochemical or even enzymatic reactions, as reported in previous studies for PS biodegradation (Tsochatzis et al., 2021a, 2021b). Benzaldehyde is also a product that can be related to styrene, as it might be generated by oxidation of styrene (Luo et al., 2023). All the remaining compounds are styrene oligomers (1,4-diphenyl-1,3-butadiene and 1,2-diphenylcyclopropane) that can be considered as PS polymer degradation products (Tsochatzis et al., 2020a) and potentially NPs (Tsochatzis et al., 2024). Moreover, another identified compounds was 2,4-di-tert-butylphenol (2,4-DTBP), which is a typical degradation product of an antioxidant additive (Irgafos 168) extensively used in polyolefins (polyethylene, polypropylene) (Su et al., 2021; Tsochatzis et al., 2020b). Finally, nonanal was also identified, which was already indicated to be a non-intentionally added substance (NIAS) in expanded PS (EPS) by Song et al. (2019). All the identified compounds that are either related to styrene or they are styrene oligomers, are given in Fig. S3 (Supporting Information).

### 3.5 Multivariate analysis

The results collected from all controls and blanks used for the digestion and absorption experiments, together with all permeability fractions (apical, cells and basolateral) were analysed for the identified compounds as listed in Table 1. Moreover, during evaluations, PS NPs/MPs size dimensions (0.06 μm and 1.0 μm) were also considered in the analysis.

An OPLS-DA focusing on the apical fraction indicated a very good fitting with a R^2^Y of 0.878 and a Q^2^ model fit of 0.859 (Fig. S4). In addition, permutation plots and CV-ANOVA verified the good fit of this model (see Fig. S5A and Table S1, Supporting Information). Interestingly, DEHP and 2,4-DTBP were also found in the blank samples. DEHP is a known phthalate, whilst 2,4-DTBP is a typical compounds resulting as degradation product from polyolefins’ antioxidant additive, asdescribed before; this points to the presence of these compounds also in the inserts used for the cell culture experiments. Compounds with a value for the variable importance in the projection (VIP) higher than 1, were DEHP and 2,4-DTBP from the blanks as well as styrene, 1,2-diphenylcyclopropane and 1,4-diphenyl-1,3-butadiene from the PS micro/nanoplastics (see Fig. S5B, Supporting Information).

Analysis of the cell fraction also indicated a good discrimination and good model fitting (Fig. S6; R^2^Y = 0.505 and Q^2^ = 0.283), although lower than in the case of the apical fraction. In addition to the apical fraction results, nonanal was also identified. Nonanal was a contaminant which came from the initially tested plastic PS (Song et al., 2019) and eventually leached into the cell system. Therefore, it is important to consider that not only MPs or NPs materials can lead to any effects, but also chemical compounds coming from the plastic material can penetrate cell membranes and have a synergistic effect. Furthermore, 1-phenylethanol was also identified, which was also reported to result from degradation of PS (Tsochatzis et al., 2021b), whilst isophthalaldehyde identified together with 1-phenyl-1,2-ethanediol (styrene-related compound) also identified to be present. Most likely, isophthlaldehyde came from the initial PS plastic as an impurity. Another compound that was found to be present in blank, analysed cell and basolateral samples was caprolactam, which is a regulated product to be used in plastic materials (European Commission, 2011), a monomer of nylon 6. Certain filters and bottles for solvent storage or PBS use nylon 6, which can be tentatively considered as the source of caprolactam in the blanks. Caprolactam is a regulated product to be used in plastic materials and its abundance indicated the presence at low (compliant) levels (low abundance), certainly below the regulated 15 ppm (European Commission, 2011). Finally, styrene was also present and clearly discriminated against PS-treated samples from the blanks together with 1,2-diphenylcyclopropane (see Fig. S6, Supporting Information). The latter was found in relatively low abundance (low signal), probably due to the low concentrations or due to the experimental conditions (exposure time, sample preparation). VIP values were identified to be higher than 1, in case of all the analytes, except in case of 1-phenyl-1,2-ethanediol. However, although model fit was good, CV-ANOVA indicated no statistical significance (p > 0.05). All MVA analysis, VIP plots and CV-ANOVA are provided in Table S2 (Supporting Information), and Fig. S7A and S7B (Supporting Information).

Although the OPLS-DA for the basolateral fraction was not statistically significant between samples, although with R^2^Y = 0.901 and Q^2^ = 0.891, an important finding of this work is that the basolateral fraction indicated the transfer of styrene as well styrene-related compounds, namely benzaldehyde, 1,2,-diphenylcyclopropane and 1,4-diphenyl-1,3-butadiene (Fig. S8). All styrene and styrene oligomers were previously reported to be of toxicity class Cramer III (Tsochatzis et al., 2020a). In addition, other NIAS such as nonanal and a phthalate (bis-2-methyl) phthalate were also found in the basolateral fraction. All MVA analysis, VIP plots and CV-ANOVA are provided in Table S3, and Fig. S9A and S9B (Supporting Information). Another compound that was found to be present in both blank, analysed cell and basolateral samples was caprolactam, which was described before together with the potential source of its presence. As in case of all fractions, the degradation product 2,4-DTBP from Irgafos 168 was again present. 2,4-DTBP presence could be explained by the fact that all cell culture methodologies are performed in multi-well plates made of plastic material, which in this work was confirmed that were made of polypropylene (PP). Therefore, 2,4-DTBP was identified to result from the blank cell culture plates.

In terms of particle size differences, there was no clear effect, and it was not possible to discriminate between the digesta containing PS particles of either 0.06 μm or 1.0 μm (NPs and MPs range), as shown in Fig. S8. The model was statistically significant and all identified molecules, as reported above had a VIP higher than 1. Therefore, it can be concluded that the PS particle size did not contribute to any difference in absorption of the chemical compounds related to the PS particles.

Finally, and in relation to the results of the three different tested fractions (see Figs. S4, S6, and S8, Supporting Information), the results between the apical and basolateral fractions were also compared (see Fig. 4). The model indicated relatively low fitting (R^2^Y = 0.444 and Q^2^ = 0.364). However, and very importantly, the model indicated that there are compounds in different intensities and abundances that pass from the apical fraction and are found to be present in the basolateral one, either styrene or styrene-related compounds. Nevertheless, it cannot be underscored that exposure times and volumes were rather limited and low, and further studies would be needed, in combination with more quantitative data.

By comparing the differences between apical and basolateral fractions (see Fig. 4, Fig. S10 and Table S4, Supporting Information), there was a clear discrimination of both fractions from the blanks, highlighting that the presence of PS in the digesta posed a significant effect. Thus, apical fraction content and results seemed to be closer to the blanks compared to basolateral, which were clearly discriminated. However, another important outcome was the fact that leached chemical compounds, and especially monomers (styrene) and oligomers (1,2-diphenylcyclopropane, 1,4-diphenyl-1,3-butadiene), as well as other compounds structurally related to monomers (benzaldehyde) among additional chemicals present in the plastic, either intentionally or non-intentionally (phthalates, nonanal) passed through cell membranes. Although no effects were identified in cell viability, the presence of these compounds indicated potential effects considering exposure time and concentrations. However, for the latter, further and more explicit studies will be needed. Moreover, for cell testing methodologies, the very small volumes can be considered as a drawback for the identification of certain chemical compounds. Another significant observation was that the use of plastic material in cell cultures needs to be addressed in such studies, as it may create some confounding effects.

## 4 Conclusions

In conclusion, our study demonstrated the effect of different sized PS NP (60 nm) and MP (1 μm) in Caco-2 HT-29/MTX differentiated cell monolayers. The polystyrene short-term exposure did not affect the viability of Caco-2 cells. Further studies should include longer exposure times and physiological polystyrene concentrations tested in cells to further investigate the potential effect on human health. The addition of PS did not alter WPI digestion, as WPI was fully digested under all conditions. Furthermore, WPI had no effect on cytotoxicity or monolayer integrity when comparing SGID WPI-PS and PS samples. The formation of a protein or protease corona on PS particles during SGID can tentatively support these findings. Untargeted GC-MS analysis confirmed the presence and transfer of various chemical compounds, including styrene, styrene-related oligomers, as well as other molecules contaminants present in the original plastic. Analysis of the basolateral fraction indicated a notable transfer of these compounds through cell membranes. DEHP and 2,4-DTBP were identified in blanks, suggesting contamination from laboratory labware materials as it was also the case for caprolactam, a regulated nylon 6 monomer. Despite no significant effects on cell viability, the findings highlighted the potential risks associated with the release of chemicals from plastics. Cell testing indicated that exposure time and test volumes may be a limiting factor, warranting further investigation. Notably, PS particle size (0.06 μm vs. 1.0 μm) did not significantly influence compound transfer as they did not pose differences in the leaving of the aforementioned identified chemicals. Therefore, the exposure to MP/NP is not related only to the micro-/nano-sized plastic but it is also related to the exposure to leaching chemicals from it.

## CRediT authorship contribution statement

**Elena Arranz:** Conceptualization, Supervision, Investigation, Methodology, Data curation, Formal analysis, Writing – original draft, Writing – review & editing, Project administration. **Emmanouil D. Tsochatzis:** Conceptualization, Supervision, Investigation, Methodology, Data curation, Formal analysis, Writing – original draft, Writing – review & editing, Project administration. **Negin Hashemi:** Investigation, Methodology, Formal analysis, Writing – review & editing. **Hanne Søndergaard Møller:** Investigation, Methodology, Formal analysis, Writing – review & editing. **Milena Corredig:** Conceptualization, Supervision, Writing – original draft, Writing – review & editing, Data curation, Formal analysis, Project administration, Resources.

## Disclaimer

The manuscript does not represent nor meant to represent an EFSA (European Food Safety Authority) opinion.

## Declaration of competing interest

The authors declare that they have no known competing financial interests or personal relationships that could have appeared to influence the work reported in this paper.

## Data Availability

Data will be made available on request.
