# Supplementary material for: Following the fate of polystyrene micro and nanobeads during in vitro digestion
Source: Curr Res Food Sci. 2025 May 19;10:101086. doi: 10.1016/j.crfs.2025.101086 (PMC12152567; doi:10.1016/j.crfs.2025.101086)
Supplement: Multimedia component 1 [file mmc1.docx]

**SUPPORTING INFORMATION**

***In vitro* testing, toxicity assessment and metabolic fate of polystyrene microplastics**

Elena Arranz^1,†^, Emmanouil Tsochatzis^2,3,†,a^, Negin Hashemi^2,3^, Hanne Søndergaard Møller^2,3^, Milena Corredig^2,3^

**Figure S1.** Cell viability (%) of confluent Caco-2 cells after 4 h incubation with 1:16 diluted (sample:DMEM complete medium) digested (SGID) formulations containing 500 μg/mL of 1 μm or 60 nm polystyrene latex particles, with or without whey protein isolated (WPI). Control cells were grown in DMEM complete medium with no treatment (100% viability).

**Figure S2.** Transepithelial electrical resistance (TEER, Ω*cm^2^) after 4 h treatment of 21 days Caco-2/HT-29/MTX co-cultures with samples containing 500 μg/mL of 1 μm or 60 nm polystyrene latex particles. Digested samples (SGID) and control H_2_O were diluted 1:16 (sample:DMEM complete medium) before cell exposure. PBS buffer was also included as control with no treatment.

**
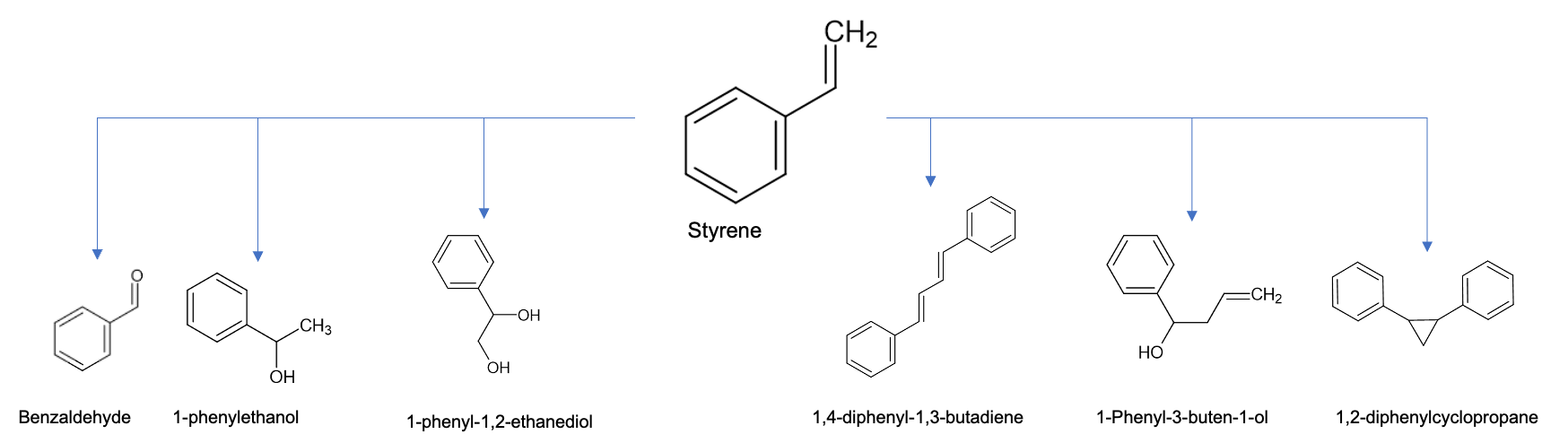
**

**Figure S3.** Identified chemical compounds structural relevant chemical to styrene.


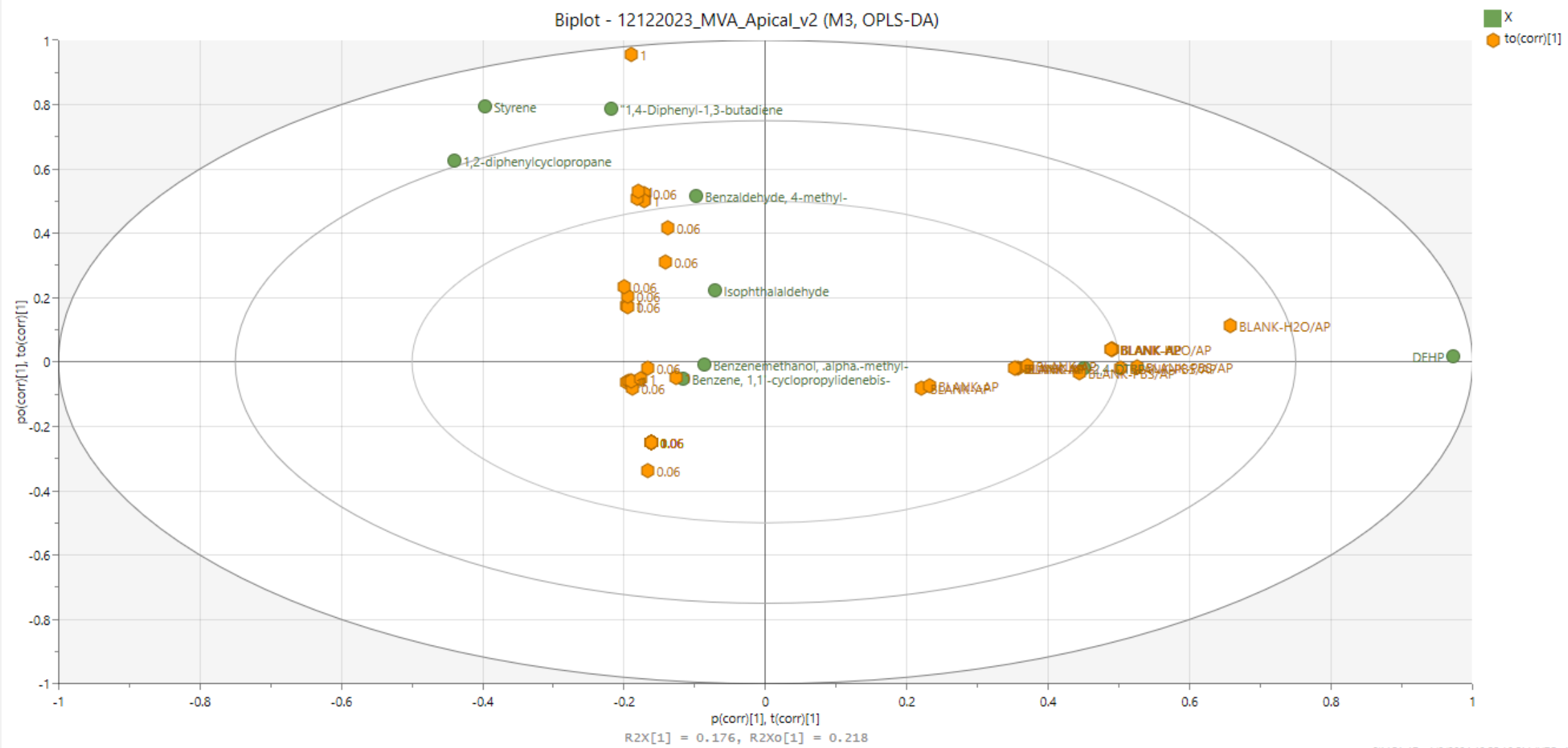


**Figure S4.** OPLS-DA biplot for the different treatments in the analysed apical fraction for the identified styrene and styrene-based compounds.

**
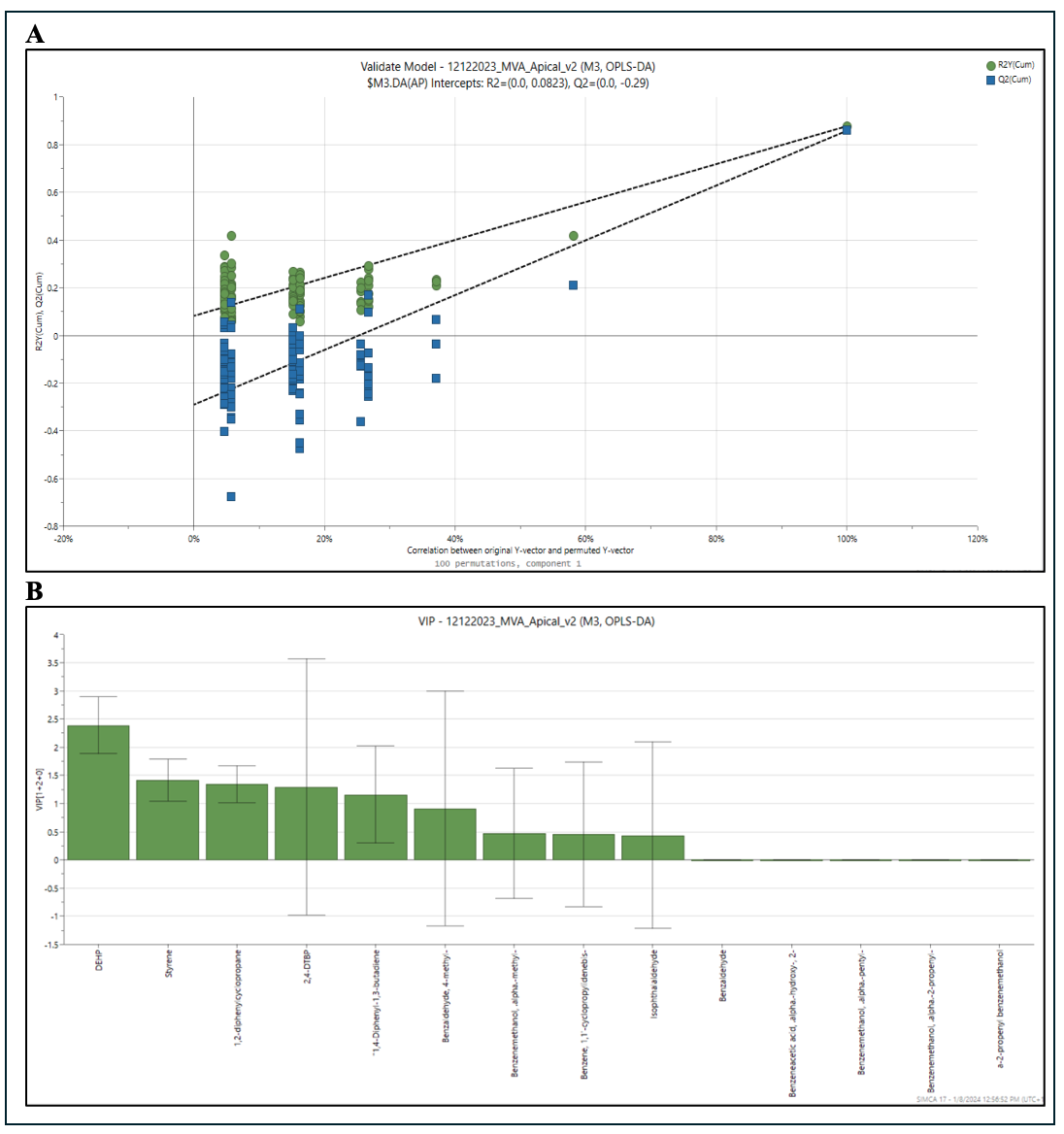
**

**Figure S5.** Permutation plot (A) for the applied OPLS-DA model for the apical fraction. Variable importance prediction (VIP) scores (B) for the selected OPLS-DA model for the apical fraction


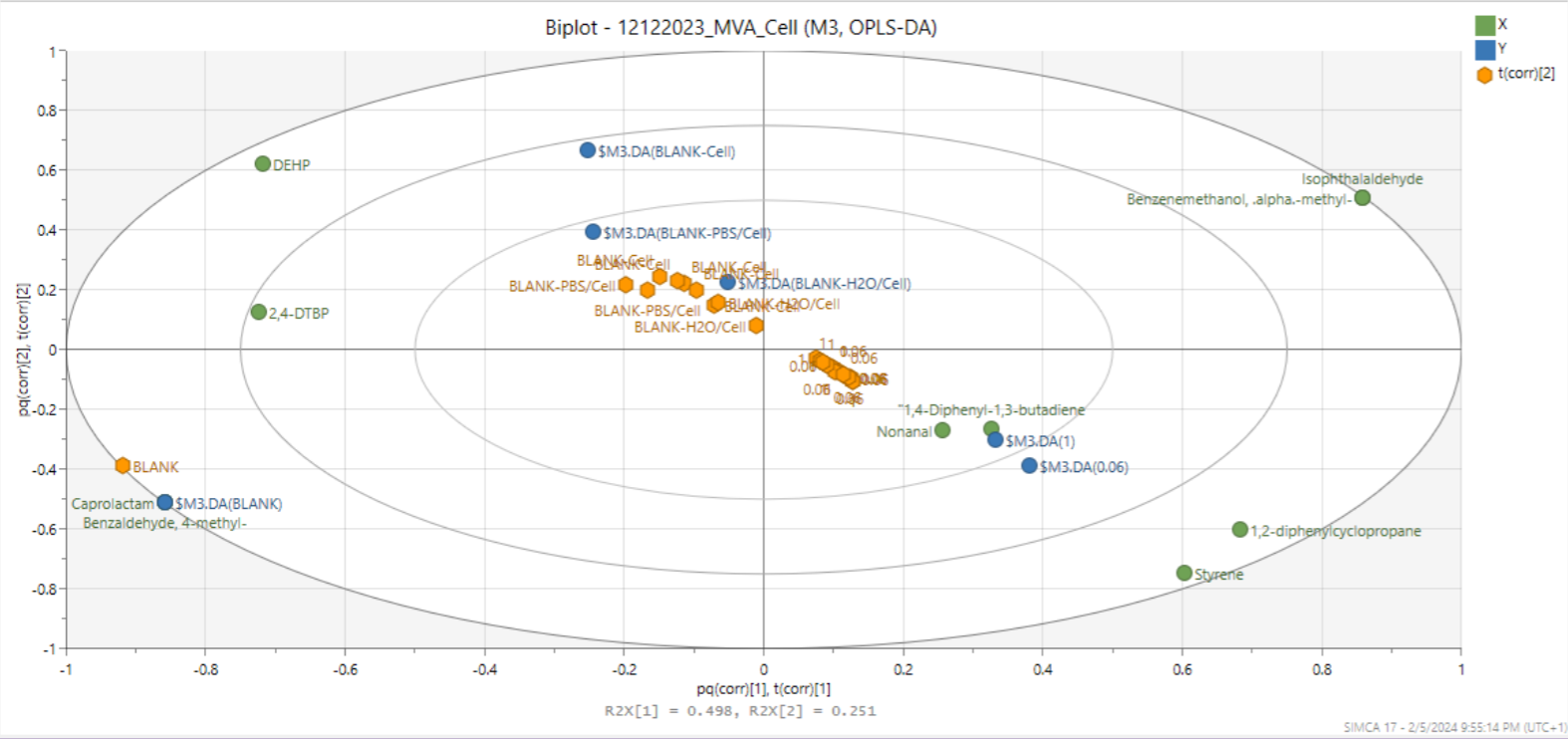


**Figure S6.** OPLS-DA biplot for the different treatments in the analysed cell fraction for the identified styrene and styrene-based compounds.

**
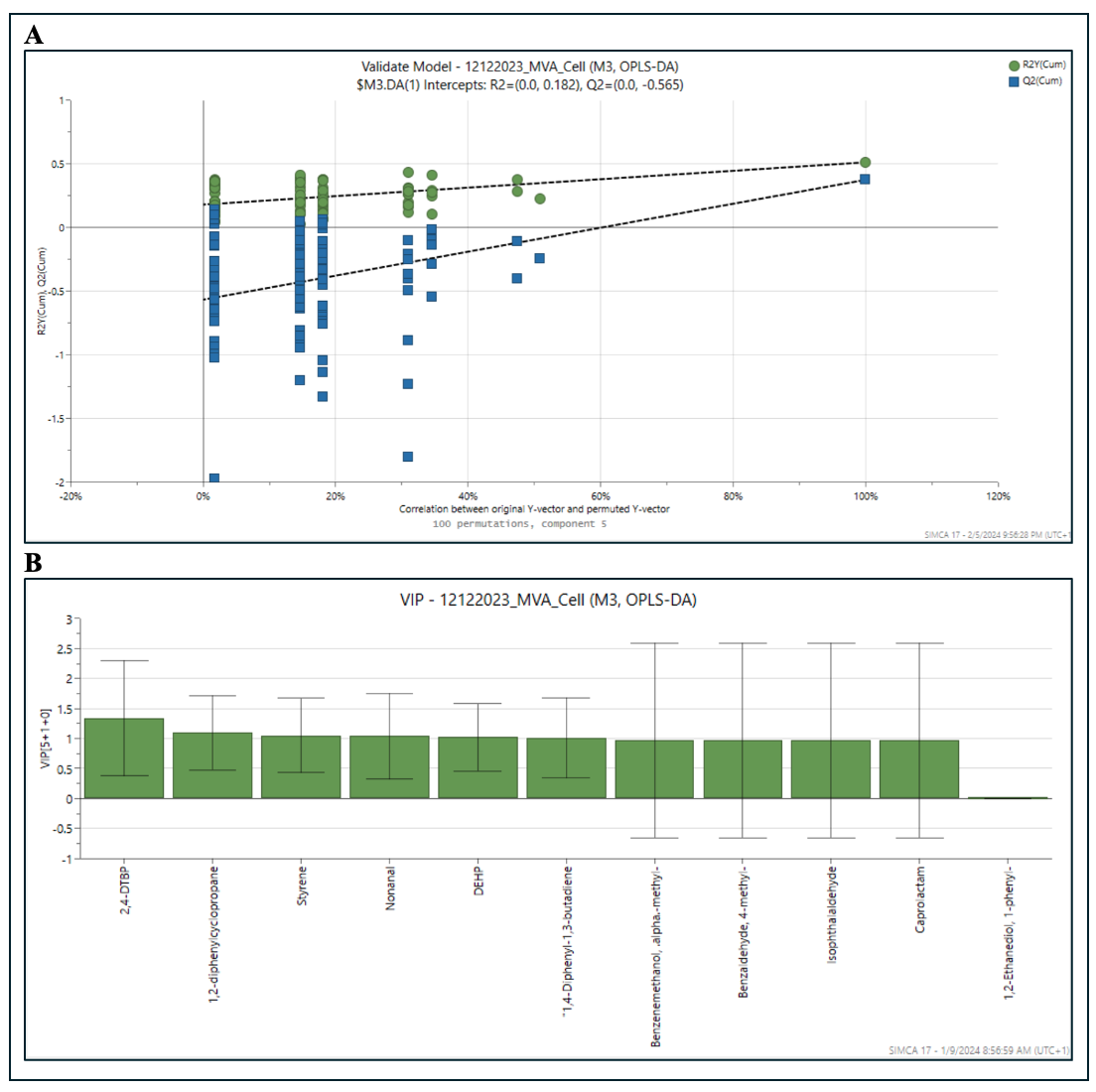
**

**Figure S7.** Permutation plot (A) for the applied OPLS-DA model for the cell fraction. Variable importance prediction (VIP) scores (B) for the selected OPLS-DA model for the cell fraction


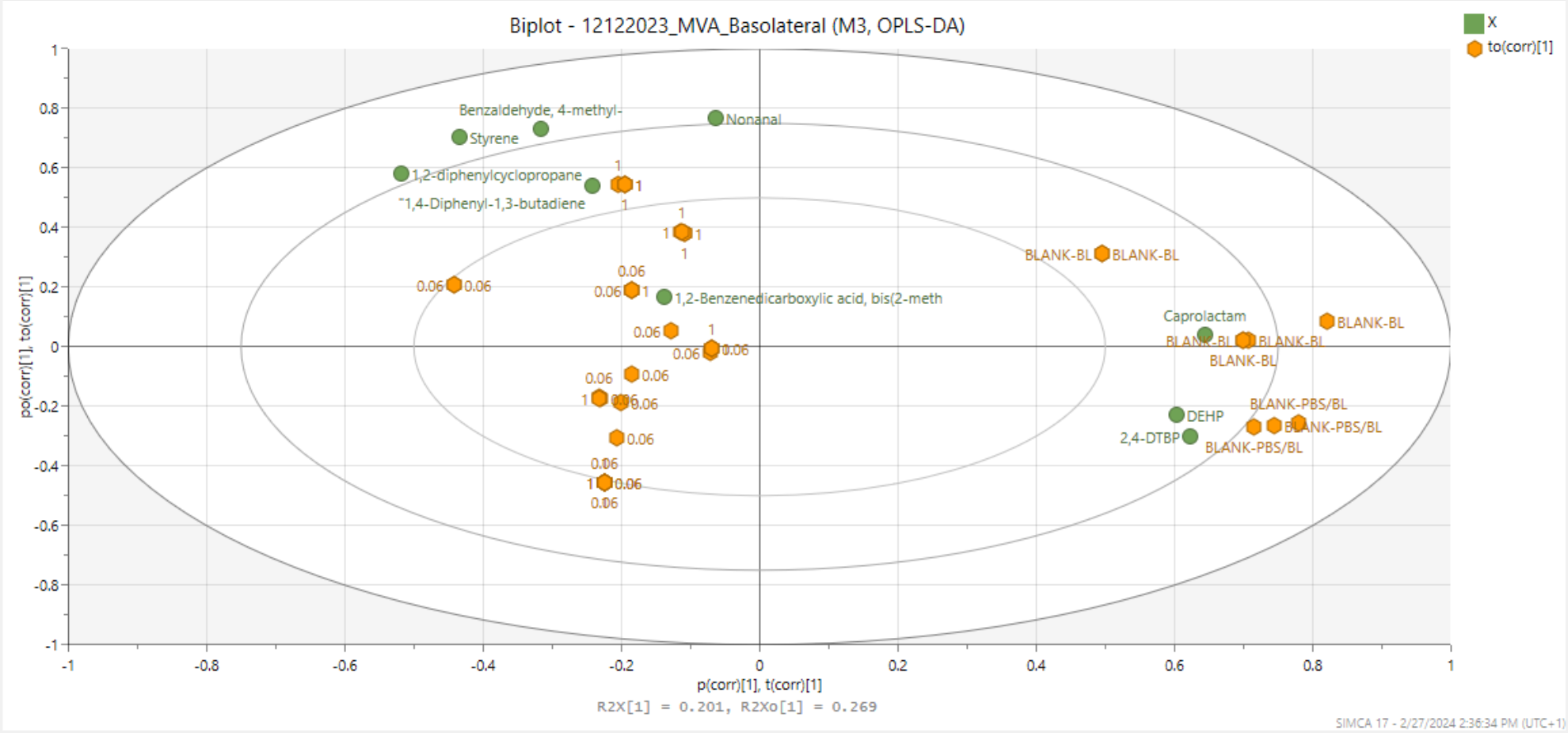


**Figure S8.** OPLS-DA biplot for the different treatments in the analysed basolateral fraction for the identified styrene and styrene-based compounds.

**
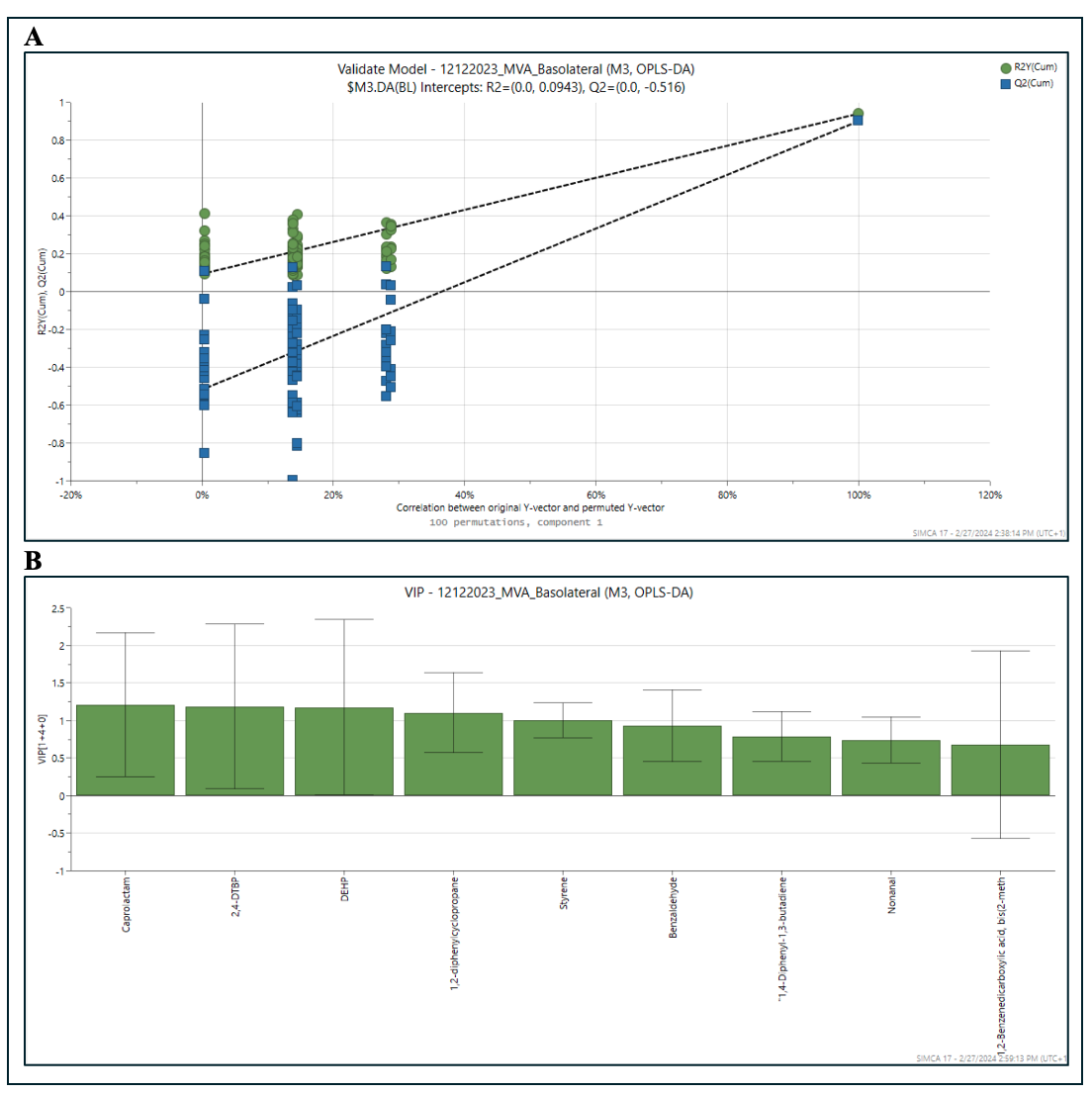
**

**Figure S9.** Permutation plot (A) for the applied OPLS-DA model for the basolateral fraction. Variable importance prediction (VIP) scores (B) for the selected OPLS-DA model for the basolateral fraction

**
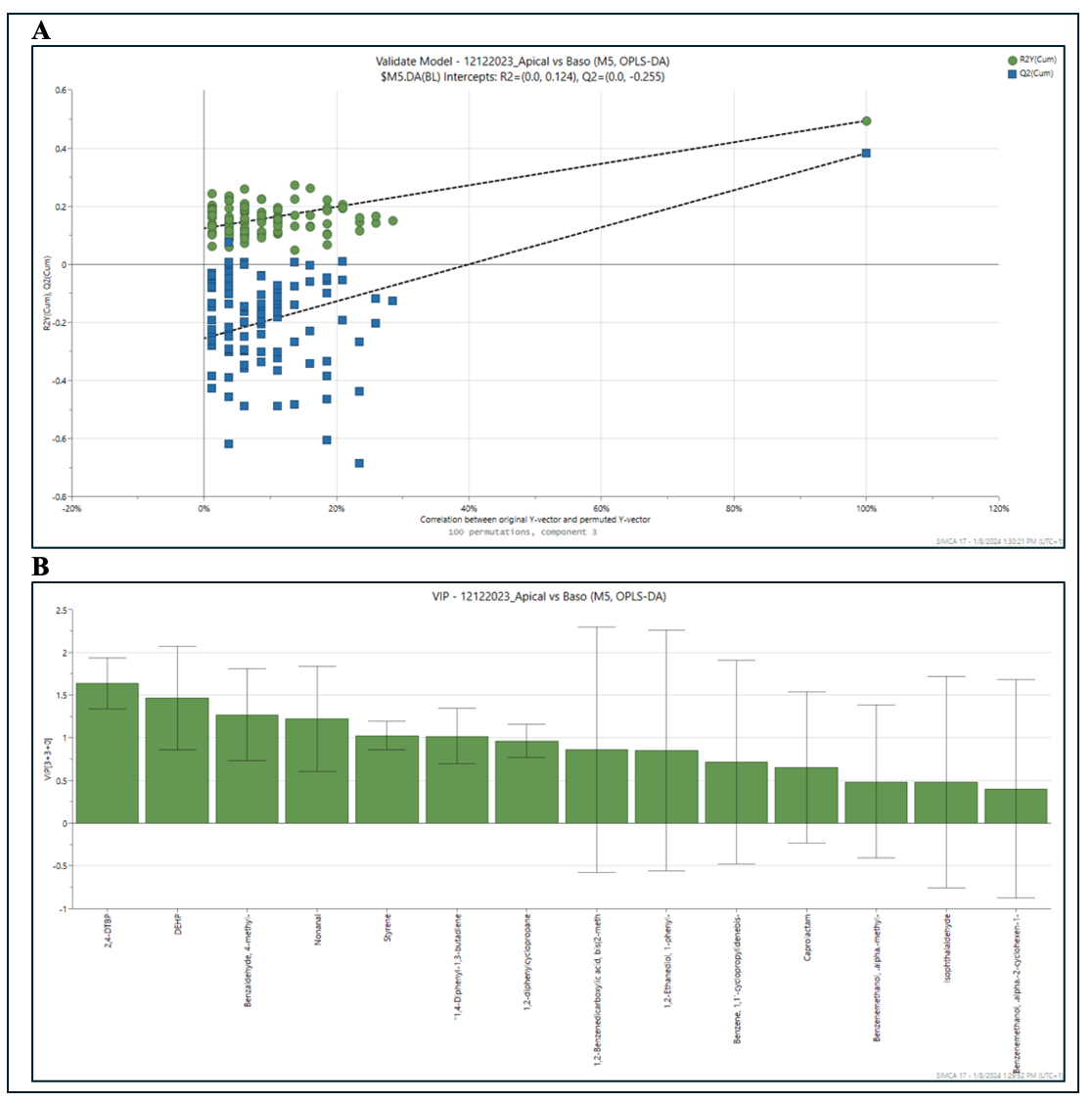
**

**Figure S10.** Permutation plot (A) for the applied OPLS-DA model for the apical/basolateral fraction. Variable importance prediction (VIP) scores (B) for the selected OPLS-DA model for the apical/basolateral fraction

**Table S1.** CV ANOVA results for the applied OPLS-DA model in case of apical fraction.

| **Model** | **SS** | **DF** | **MS** | **F** | **P** | **SD** |
| --- | --- | --- | --- | --- | --- | --- |
| **Total corr.** | 48 | 48 | 1 |  |  | 1 |
| **Regression** | 42.2152 | 6 | 6.8692 | 42.5223 | 2.71328e-16 | 2.62092 |
| **Residual** | 6.78482 | 42 | 0.161543 |  |  | 0.401925 |

**Table S2.** CV ANOVA results for the applied OPLS-DA model in case of cell fraction.

| **Model** | **SS** | **DF** | **MS** | **F** | **P** | **SD** |
| --- | --- | --- | --- | --- | --- | --- |
| **Total corr.** | 135 | 135 | 1 |  |  | 1 |
| **Regression** | 51.2108 | 55 | 0.931105 | 0.888998 | 0.675817 | 0.964938 |
| **Residual** | 83.7892 | 80 | 1.04737 |  |  | 1.02341 |

**Table S3.** CV ANOVA results for the applied OPLS-DA model in case of basolateral fraction.

| **Model** | **SS** | **DF** | **MS** | **F** | **P** | **SD** |
| --- | --- | --- | --- | --- | --- | --- |
| **Total corr.** | 40 | 40 | 1 |  |  | 1 |
| **Regression** | 36.1013 | 10 | 3.61013 | 27.7796 | 1.79845e-12 | 1.90003 |
| **Residual** | 3.89869 | 30 | 0.129956 |  |  | 0.360494 |

**Table S4.** CV ANOVA results for the applied OPLS-DA model in case of apical/basolateral comparison.

| **Model** | **SS** | **DF** | **MS** | **F** | **P** | **SD** |
| --- | --- | --- | --- | --- | --- | --- |
| **Total corr.** | 246 | 246 | 1 |  |  | 1 |
| **Regression** | 133.104 | 36 | 3.69734 | 6.87749 | 2.63432e-20 | 1.92285 |
| **Residual** | 112.896 | 210 | 0.5376 |  |  | 0.733212 |
